# 

**Published:** 1909-07

**Authors:** 


### INDEX.

Abstracts and Selections....23, 70, 106, 154, 197, 237, 275, 320, 362, 394, 436 .... 580
A Brief Discussion of the Economic Importance of the Most Common Adult Cestodes of Man in the United States (Taenia Saginata, Dibothriocephalus latus, Hymenopelis Nana, and Taenia Solium); With Report of Two Cases. By Charles A. Pfender, M. D., Washington, D. C........ 293
A Commendable Reform.... 102
Asthenopia Due to Disorders of the Stomach. By H. L. Hilgartner, M. D., Austin, Texas.... 254
Atropine. By H. H. Redfield, M. D.... 251
Bichloride of Mercury Baths in the Treatment of Variola. By R. H. L. Bibb, M. D., Saltillo, Mexico.... 43
Books and Magazines...........37, 159, 242, 332, 407, 452, 586
Causes of Insanity.... 417
Chronicles of the Octopus.... 220
Correspondence............................192, 233, 319, 359
Delirium Tremens—A New Plan of Treatment. By Geo. E. Pettey, M. D., Memphis, Tenn.... 256
Diagnosis and Surgical Treatment of Acute Pancreatitis. By Emory Lanphear, M. D., Ph. D., LL. D., St. Louis, Mo.... 209
Doctor as a Missionary and Apostle of Hygiene, The.... 427
Dr. James W. McLaughlin.... 213
Echoes of the Dallas Meeting.... 573
Editorialets........20, 67, 104, 149, 189, 229, 271, 318, 357, 430
Elements of Decay in American Civilization. By F. E. Daniel, M. D., Austin, Texas.... 1
Emetine. By George L. Servoss, M. D., Fairview, Nevada.. 143
Ergotin. By H. H. Redfield, M. D., Chicago, Ill.... 385
Fights of the Majority, The.... 183
Fly as the Main Distributor of Typhoid Fever, The. By Addison L. Lincecum, M. D., Louise, Texas.... 178
Functional Heart Disease. By Geo. F. Butler, M. D.... 340
Great Medical Association of the Southwest to Meet Next Month in San Antonio.... 148
Hon Jewel P. Lightfoot.... 217
Hookworm and Pellagra.... 266
Imperial Caesar Redivivus.... 186
Lebelin. By George L. Servoss, M. D., Fairview, Nevada.... 337
Letters.... 273
Life, Character and Works of Prof. J. W. McLaughlin, M. D. By H. L. Hilgartner, M. D., Austin, Texas.... 557
Machine Methods of Defense. By G. Frank Lydston, M. D., Chicago, Ill.... 263
Not a thing to Wear. By W. A. Philpott, Jr.... 145
"Now You See It. Now You Don't. Who Can Tell Where the Little Joker Is?".... 428

Nuclein in Tuberculosis. By James Alexander, M. D., Bennett Medical College, Chicago .......... 335
Observations on Tubercular Hip Joint Diseases. By L. Sexton, M. D., New Orleans, La .......... 258
Obstetrical Notes and Comments. By W. T. Marrs, M. D., Peoria, Ill .......... 380
Our Congested Asylums .......... 267
Our Duty in Relation to Alcohol. By John M. Shaller, M. D., Denver, Colo .......... 567
Placenta Praevia. By B. W. Skipper, M. D., Lovelady, Texas. 94
Psychology of Drunkenness. By Eloise Shepherd (Proserpine), Houston, Texas .......... 9
Publisher's Department......34, 80, 124, 162, 204, 244, 286, 327, 370, 409, 453 .......... 587
Rally on the Great M. V. at St. Louis .......... 101
Resolutions Adopted by the Chicago Medical Society, January 11, 1910. (The A. M. A.) .......... 315
Regulation of Marriage to Cut Down Ratio of Insanity ...... 375
Russianizing of the Medical Profession by the Political Machine of the A. M. A. By G. Frank Lydston, M. D., Chicago, Ill .......... 127
Sanguinarine. By George L. Servoss, M. D., Fairview, Nevada 313
Selected .......... 343
Some Cases from My Note-Book of Clinical Experience. By A. W. Fly, M. D., Galveston, Texas .......... 564
Some Remarks on the Pathogenesis of Tuberculosis. By Theo. Y. Hull, B. S., M. D., San Antonio, Texas .......... 85
Some Unusual Cases. By A. W. Fly, M. D., Galveston, Texas. 16
Song of the First Lord .......... 225
Sterilization of Male Insane .......... 61
Suit against the American Medical Association for Damages.. 19
Summer Diseases of Children. By William Edward Fitch, M. D., New York City .......... 47
Surgical Drainage. By L. Sexton, M. D., New Orleans, La... 421
The Dictator of Medical Ethics .......... 219
The Emancipation from Tuberculosis. By Theo. Y. Hull, B. S., M. D., San Antonio, Texas .......... 309
The Fly in the Ointment .......... 226
The House Fly as a Disease Carrier. By Alexander S. Garrett, M. D., Springtown, Texas .......... 136
The Journal's Own Page .......... 366
The Lifting of the Veil .......... 227
The Physician as a Temperance Reformer. By Alexander S. Garrett, M. D., Springtown, Texas .......... 55
The Russianizing of the Medical Profession by the Political Machine of the A. M. A. By G. Frank Lydston, M. D., Professor of Genito-Urinary Surgery in the State University of Illinois, Medical Department, Chicago .......... 169
Tonsilitis. By Charles J. Drueck, M. D., Chicago, Ill...... 387
Treatment of Pellagra .......... 147

